# Disinfection of methicillin-resistant *Staphylococcus aureus*, vancomycin-resistant *Enterococcus faecium* and *Acinetobacter baumannii* using Klaran WD array system

**DOI:** 10.1099/acmi.0.000194

**Published:** 2021-09-15

**Authors:** Richard M. Mariita, Rajul V. Randive

**Affiliations:** ^1^​ Crystal IS, Inc., an Asahi Kasei company, 70 Cohoes Avenue, Green Island, New York, 12183, USA

**Keywords:** *Acinetobacter baumannii*, disinfection, healthcare-associated infections, methicillin-resistant *Staphylococcus aureus *(MRSA), UVC LEDs, vancomycin-resistant enterococcus faecium, HVAC

## Abstract

Hospital-associated infections (HAIs) are a major burden in healthcare systems. In this study, UVC LEDs emitting radiation from 260 to 270 nm were evaluated for effectiveness in reducing methicillin-resistant *Staphylococcus aureus* (MRSA), vancomycin-resistant *Enterococcus faecium* and *Acinetobacter baumannii *. The array has four WD LEDs, each with 70 mW placed at 7 cm from test organisms. With 11.76 mJ cm^−2^, the study obtained 99.99% reduction (log_10_ reduction factor of 4) against MRSA and VRE. For *A. baumannii *, 9 mJ cm^−2^ obtained 99.999% reduction (log_10_ reduction factor of 5). These results present scientific evidence on how effective UVC LEDs can be used in the fight against HAIs.

## Introduction

Worldwide, hospital-acquired infections (HAIs) are responsible for extended admissions, increased medical costs, and noticeable morbidity and mortality [1]. Per year, it is approximated that in the USA alone, 2 million patients suffer from HAIs, while nearly 90 000 are estimated to die. The overall direct cost of HAIs to hospitals ranges from US $28–45 billion [2]. The micro-organisms responsible for HAIs include methicillin-resistant *Staphylococcus aureus* (MRSA), vancomycin-resistant enterococci (VRE) and *Acinetobacter baumannii.* Here, UVC LEDs emitting radiation at 260–270 nm were evaluated for effectiveness in reducing MRSA ATCC 33592, VRE ATCC 700221 and *A. baumannii* ATCC 19606.

*S. aureus* is a major pathogen both within hospitals and in the community, as healthcare systems in the USA and Europe have seen the prevalence of MRSA increase from <3% in the early 1980s to rates as high as 40% in the 1990s [3]. In 2017 alone, the USA had 119 000 infections and almost 20 000 deaths [4]. MRSA costs ~US $10 billion a year to treat in the USA, averaging ~US $60 000–70 000 per patient [5]. MRSA can be transmitted via surgical tools, high-touch surfaces, air in surgical units as well via intubation in adult intensive care unit (ICU), causing pneumonia [3].

VRE is of medical importance, as it is associated with serious multidrug-resistant infections [6]. Moreover, in the USA, the emergence of VRE in water is threatening the health of human beings due to antibiotic resistance [6] and the life-threatening nature of some VRE infections [6]. VRE has been isolated from most sites and objects in healthcare facilities, including medical equipment (ventilator tubing, pumps, wash bowls, automated medication dispensers, intravenous poles), monitoring devices (call bells, electrocardiographic monitors, pulse oximeters, glucose meters, stethoscopes, electronic thermometers, blood pressure cuffs, keyboards, wall-mounted control panels), furniture (telephones, air cushions, headboards, tables, chairs, bed rails), toilet seats, doors, floors and linens [7]. In the USA, hospital costs due to VRE infections vary between US $9949–77 558 [8].

Globally, *A. baumannii* is one of the most clinically significant multidrug-resistant organisms [9]. *A. baumannii* can be aerosolized, and has been found to contaminate trauma ICUs and HVAC systems, especially in locations with high humidity, causing pneumonia and urinary tract and bloodstream infections [9]. In 2017, carbapenem-resistant *Acinetobacter* caused an estimated 8500 infections in hospitalized patients and 700 estimated deaths in the USA alone [10] with US $281 million in healthcare costs. There are few treatment options, hence the designation of *A. baumannii* as a pathogen of urgent concern and priority [10].

### Study objectives

Given the economic and health challenges associated with HAIs, additional effective strategies for disease prevention are needed. The objective of this study was to evaluate the disinfection performance of a UVC LED array against three causative agents responsible for HAIs. Specifically, the study investigated the UVC dose required for inactivation of MRSA (ATCC 33592), Enterococcus faecium (ATCC 700221) and *A. baumannii* (ATCC 19606). These results will be used as a baseline in determining the UVC dose required in the control of causative agents responsible for HAIs, thus minimizing extensive use of antibiotics.

## Methods

### Strain and culture conditions

All strains were stored at −80 °C in appropriate culture media using sterile 20 % glycerol stock. Working strain cultures were stored at 4 °C and propagated before being used in UVC disinfection tests. MRSA and *A. baumannii* strains were propagated in ATCC Medium 3 (nutrient agar/broth), whereas VRE was propagated in ATCC Medium 44 (brain heart infusion agar/broth). Cultures were incubated for 18–20 h at 37 °C with shaking at 180 r.p.m. For use, each strain was harvested by centrifugation at 4000 r.p.m. for 10 min. The pellets were washed using 1× phosphate-buffered saline (PBS) three times for 10 min. Between each wash, the supernatant was discarded, and the remaining pellet was resuspended by vortexing. After being washed thrice, the pellet was resuspended in 1× PBS used for static dosing study. Agar plates containing irradiated strains were incubated at 37 °C for a further 18–20 h. Static dosing was performed in three independent replicates and means were used in statistics.

### UVC LED disinfection

The UVC disinfection efficiency of a 4 UVC LED array was evaluated using stationary growth phase bacteria. The array emitted radiation at a wavelength of 260–270 nm, as confirmed using an Ocean Optics USB4000 photospectrometer. The distance between the UVC LEDs and agar plate inoculated with bacteria suspension was 7 cm. Disinfection efficacy was assessed after 3, 6, 9, 12 and 15 s of irradiation (Table 1). Controls were not UVC dosed. Log reduction value (LRV) was calculated using the equation:

$$LRV=log_{10}\;(\frac{A}{B})$$

**Table 1.** Effectiveness of UVC disinfection against methicillin-resistant *Staphylococcus aureus* (MRSA), vancomycin-resistant *Enterococcus faecium* and *Acinetobacter baumannii* at 7 cm using an array with 4 UVC LEDs. Experiments were performed in triplicate and data are expressed as means

Strain	UVC dose (mJ cm^−2^)	Irradiation time (seconds)	Control c.f.u. ml^−1^ (−UVC)	Test c.f.u. ml^−1^ (+UVC)	LRV
VRE	3.15	3	1.00E+09	1.11E+07	1.96
VRE	6.18	6	1.00E+09	5.43E+06	2.26
VRE	9.00	9	1.00E+09	6.67E+05	3.18
VRE	11.76	12	1.00E+09	5.57E+04	4.25
VRE	16.5	15	1.00E+09	No growth	–
MRSA	3.15	3	1.61E+08	1.10E+07	1.17
MRSA	6.18	6	1.61E+08	1.00E+06	2.21
MRSA	9.00	9	1.61E+08	1.33E+05	3.08
MRSA	11.76	12	1.61E+08	1.00E+04	4.21
MRSA	16.5	15	1.61E+08	No growth	–
* A. baumannii *	3.15	3	2.49E+07	3.55E+03	3.85
* A. baumannii *	6.18	6	2.49E+07	2.30E+02	5.03
* A. baumannii *	9.00	9	2.49E+07	8.67E+01	5.46
* A. baumannii *	11.76	12	2.49E+07	2.53E+01	5.99
* A. baumannii *	16.5	15	2.49E+07	3.33E+00	6.87

where *A* is c.f.u. ml^−1^ for UVC controls (−UVC) and *B* is c.f.u. ml^−1^ for UVC dosed (UVC+).

To determine the relationship between irradiation time and disinfection efficacy against each test strain, linear regression analysis was performed using GraphPad software (https://www.graphpad.com/).

## Results

**Fig. 1.**
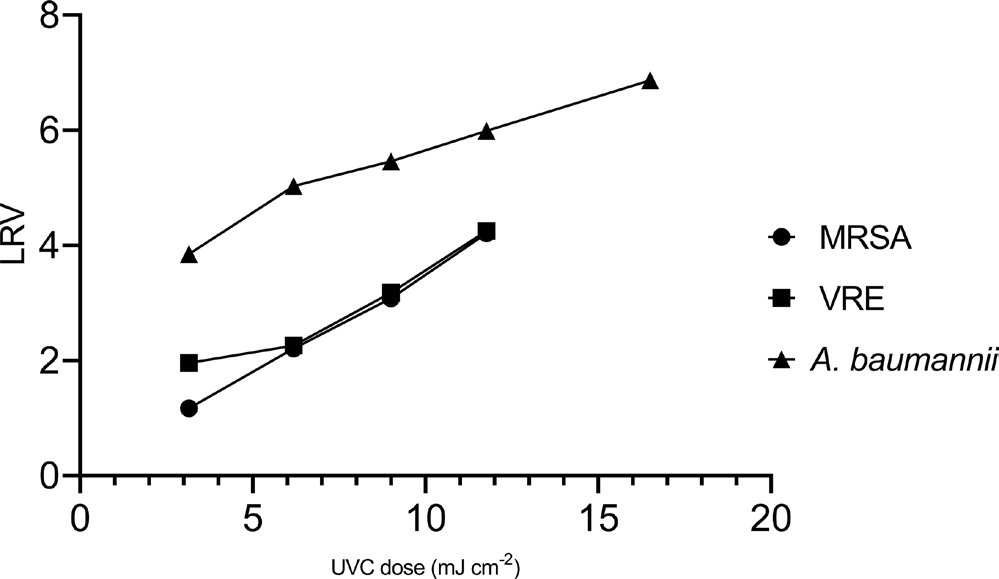
Disinfection efficacy of UVC against key pathogens associated with HAIs. *A. baumannii* was more sensitive against UVC compared to MRSA and VRE.

Table 1 shows that the UVC LED array is very effective against the study test strains (MRSA ATCC 33592, VRE ATCC 700221 and *A. baumannii* ATCC 19606). The study revealed that 12 seconds of irradiation time yielded 11.76 mJ cm^−2^ UVC dose, which was enough to obtain >LRV4 (99.99% reduction) against the three test strains (Table 1). Additionally, the study revealed that *A. baumannii* was more susceptible to UVC radiation than MRSA and VRE strains (Fig. 1).

Linear regression analysis at 95% for MRSA (*R* ^2^=0.9969, *P*=0.0016), *A. baumannii* (*R* ^2^=0.9681, *P*=0.0024) and VRE (*R* ^2^=0.9403, *P*=0.0303) displayed strong association between irradiation time and disinfection efficacy.

## Discussion

MRSA is transmitted through high-touch surfaces or direct contact with infected individuals [11]. The mode of transmission for *A. baumannii* [12] and VRE [13] is similar to that of MRSA. Disinfection of high-touch surfaces is thus an obvious intervention for infection control [14], especially in healthcare facilities to protect the already vulnerable patients. Although enhanced cleaning has been shown to reduce MRSA infections in wards [14], additional measures can be put in place in areas such as surgical rooms, where some MRSA transmission can be caused by airborne transmission [15].

This study has demonstrated in the laboratory that the use of LEDs that emit 260–270 nm UVC radiation has the capability to achieve high levels of inactivation of micro-organisms that are causative agents of HAIs. The UVC radiation acts by exerting germicidal affect on DNA, leading to breakage of molecular bonding [16]. A microbial disinfection strategy using UVC LEDs leaves no waste and, unlike mercury lamps, there is no potential exposure to mercury and there are no disposal-related hazards. The inactivation efficacy from our baseline study supports previous demonstrations that revealed that the use of UVC radiation will be a beneficial adjunct to other existing strategies, leading to further reduction of microbial agents responsible for HAIs [17].

Our study revealed that *A. baumannii* was more susceptible to UVC radiation than MRSA and VRE strains. This is possibly due to *A. baumannii* being Gram-negative [18] as opposed to MRSA and VRE, which are Gram-positive [19]. As a rule, Gram-positive bacteria are more resistant to UV compared to Gram-negative ones [20]. This can be attributed to morphological differences. For instance, the presence of thicker cell walls in Gram-positive bacteria could reduce the amount of UVC reaching the cellular DNA [21].

Based on these baseline findings, it is proposed that a further feasibility study in areas such as in intensive care and surgical units as well as other high-touch surfaces be undertaken.

### Conclusion

The results from this study suggest that arrays of Klaran WD UVC LEDs emitting radiation between 260–270 nm can provide effective and rapid decontamination of HAIs. Further *in situ* tests to assess usability and relative performance would be justified.
